# Inflammation causes arginine to become an essential amino acid in critically ill children

**DOI:** 10.1186/cc9804

**Published:** 2011-03-11

**Authors:** CT De Betue, DA Van Waardenburg, KF Joosten, NE Deutz

**Affiliations:** 1 Erasmus MC, Rotterdam, the Netherlands; 2 MUMC, Maastricht, the Netherlands; 3 UAMS, Little Rock, AR, USA

## Introduction

In critically ill children we previously found decreased plasma levels of arginine (Arg) and its precursor citrulline (Cit), with a strong inverse relation to C-reactive protein (CRP) [1]. Cit is the sole precursor of Arg *de novo *synthesis in the body. We hypothesized that Arg becomes an essential amino acid, because Cit availability is reduced during inflammation. Therefore we studied Cit and Arg production, using stable isotope technology, in relation to the severity of inflammation in critically ill children.

## Methods

Twenty-two critically ill children (age 0.89 ± 0.04 years) with different levels of inflammation were studied on day 3 post-admission; viral bronchiolitis (group 1, *n *= 9), infectious disease without shock (group 2, *n *= 6) and septic shock (group 3, *n *= 7). A 2-hour stable isotope tracer protocol was performed after at least 4 hours fasting to determine Arg and Cit kinetics. Data presented as mean ± SE. Statistics by ANOVA, Spearman's correlation.

## Results

**Figure 1:**
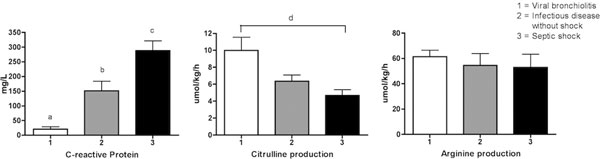
**CRP, Cit and Arg production**. CRP, *P *< 0.01 between all groups (a, b, c); d, *P *< 0.05.

See Figure 1 for results per group. CRP was significantly different between groups. Cit production was significantly lower in the group with highest inflammation compared with the group with lowest inflammation. Cit production was inversely correlated with plasma CRP (*r *= -0.58, *P *< 0.001).

## Conclusions

Our data show that with increasing rate of inflammation the production of Arg's precursor Cit is severely depressed. Previously we found that *de novo *Arg production is almost equal to Cit production [2]. As a consequence, Arg availability becomes fully dependent on tissue protein breakdown and nutrition. Inflammation causes Arg to become an essential amino acid in critically ill children.
